# Comparison of methods for isolating fungal DNA

**DOI:** 10.1016/j.plabm.2021.e00221

**Published:** 2021-04-17

**Authors:** Ilaria Galliano, Valentina Daprà, Elena Zaniol, Carla Alliaudi, Elisa Graziano, Paola Montanari, Cristina Calvi, Massimiliano Bergallo

**Affiliations:** a Department of Public Health and Pediatrics, University of Turin, School of Medicine, Turin, Italy; b BioMole Srl, Via Quarello 15/A, 10100, Turin, Italy

**Keywords:** Fungal DNA, DNA isolation, PCR, *Mucorales*

## Abstract

**Objectives:**

The main aim of this work was to compare the methods of DNA isolation in the moulds of genus *Mucorales* with special regard to the amount and purity of the DNA acquired. The acquired DNA was then amplified by specific real-time PCR.

**Design:**

Five DNA extraction procedures were carried out in a Class 2 Biosafety cabinet in a dedicated room with suitable biosafety precautions and appropriate biowaste disposal methods. A total of 6 *Mucorales* clinical strains were used.

**Results:**

From the viewpoint of concentration and purity, methods A shown abundant amount of fungal DNA whereas methods E report a pure fungal DNA with R260/280 of 1.7 near the optimal 1.8. The DNA quantity reach statistically difference at ANOVA test with p value 0.0005

**Conclusion:**

Overall, the E method was the most efficient method in the extraction of DNA from fungal cultures compared to the other methods considering time, cost, technical expertise, and instrumentation. Use of this assay will allow researchers to obtain DNA from fungi quickly for use in molecular assays

## 1 Introduction

“Mucormycosis” represents a wide spectrum of infections caused by fungi *zygomycetes,* order *Mucorales, Phylum Zygomycota*. The most common genera causing diseases are *Rhizopus, Mucor, Rhizomucor, Cunninghamella, and Lichtheimia* (formerly Absidia) [[1], [2], [3]]. The incidence of mucormycosis is relatively low, but it constitutes a significant area of concern in immunocompromised patients, including pediatric patients with malignances [4,5]. One of the major problems concerning mucormycosis is the early accurate diagnosis and the identification of the involved organism [6,7].

Nucleic acid detection methods such as PCR have become a common tool for microbial identification and diagnosis. Although PCR amplification can be performed directly for various microbial cultures, for filamentous fungi and yeasts, prior isolation of DNA is often preferred. As the DNA extraction process eliminates many unknown interfering substances present in the biological material, it plays an important role in ensuring consistent test results. Toward this end, considerable efforts have been made to enable improved DNA preparation from fungi [[8], [9], [10]]. Many of these methods rely on using a grinder (with or without liquid nitrogen) for initial breaking up of the mycelia. This is a significant handicap when dealing with a large number of samples. Since no routine tests for the mucormycosis diagnosis are currently available and DNA-based techniques for detection are not yet fully standardized or commercially available, the main aim of this work was to compare the methods of DNA isolation in the moulds of genus *Mucorales* with special regard to the amount and purity of the DNA acquired. The acquired DNA was then amplified by specific real-time PCR.

## 2 Materials and methods

The DNA extraction procedures were carried out in a Class 2 Biosafety cabinet in a dedicated room with suitable biosafety precautions and appropriate biowaste disposal methods. A total of 6 *Mucorales* clinical strains were used. These included: *3 Mucor* spp. *and 3 Rhizopus* spp. The Bacteriology and Mycology Laboratory, Public Health and Pediatrics Department, University of Torino, Italy provided all fungi. Filamentous fungi were identified according to macroscopic and microscopic morphological procedures and maintained on potato dextrose agar (PDA; Merck KGaA, Darmstadt, Germany) at 25 ​°C. Before the assays, fungi were transferred to Sabouraud dextrose (SAB; Merck KGaA) agar and incubated for 24–72 ​h, in duplicate until hyphal growth was observed [11]. Mycelial tufts were removed and suspended with 0.85% saline. After a brief vortex, the mycelial tufts were allowed to settle by keeping the tubes at room temperature for 10 ​min. The resulting suspensions were removed and vortexed thoroughly. After the settling of the larger particles, suspensions were adjusted by nephelometry and diluted in saline to obtain inocula of 2 ​× ​104 ​CFU ml-1, as confirmed by colony counts in triplicate on SAB agar [11].

For DNA extraction five named methods A, B, C, D and E protocol were compared. In brief, in A method mycelial tufts were boiled for 10min, vortexed and boiled for other 10min and then centrifuged at 14,900 ​g for 5min at 25 ​°C. 250 ​μl of supernatant were added to an equal amount of isopropanol, and incubated at −80 ​°C for 30min. After centrifugation at 14,900 ​g for 2min, the pellet was washed with 70% ethanol, centrifuged at 14,900 ​g for 5min, dried, and suspended in 20 ​μl of ultrapure H2O; in B method mycelial tufts were filtered thorugh cheese cloth and manually ground in 1.5 ​ml of microfuge tubes with micro pestle by adding 500 ​μL of TES Lysis buffer (100 ​mM TrisHCl pH 8; 10 ​mM EDTA pH8; 2% SDS) followed by microwave treatment at 800 ​W frequency in microwave oven 230 ​V output (Maxima Kitchen Equipment Nijverheidsweg 19F, ND) at 28 ​°C for 15s. incubated in 60 ​°C for 30 ​min centrifugated at 10,000 ​g/5 ​min. DNA was precipitated by adding 0.6 ​vol of ice cold isopropanol and suspended in 20 ​μl of ultrapure H2O; in C method mycelial tufts were filtered thorugh cheese cloth and manually ground in 1.5 ​ml of microfuge tubes with micro pestle by adding 200 ​μL of C Lysis buffer (100 ​mM NaCl, 10 ​mM TrisHCl ph8, 1 ​mM EDTA pH8, 1% SDS, 2% Triton X-100) followed by on incubation at −80 ​°C until it freezes (this step was repeated two times), after addition of 200 ​μL of chloroform the samples was vortex and centrifuged at 14,900 ​g for 2min. 400 ​μl of supernatant were added to an equal amount of ethanol, centrifuged and washed with 70% ethanol, centrifuged at 14,900 ​g for 5min, dried, and suspended in 20 ​μl of ultrapure H2O; in D method mycelial tufts were filtered thorugh cheese cloth and manually ground in 1.5 ​ml of microfuge tubes with micro pestle by adding 500 ​μL of β-mercaptoethanol. After incubation at room temperature 600 ​μl of chloroform was added, centrifugated at 10,000 ​g/5 ​min. DNA was precipitated by adding 1 ​vol of ice cold ethanol and the pellet was washed with 70% ethanol, centrifuged at 14,900 ​g for 5min, dried, and suspended in 20 ​μl of ultrapure H2O; in E method mycelial tufts were filtered thorugh cheese cloth and manually ground in 1.5 ​ml of microfuge tubes with micro pestle by adding 500 ​μL of A Lysis buffer (500 ​mM NaCl, 400 ​mM TrisHCl ph7.5, 50 ​mM EDTA pH8, 1% SDS) at 60 ​°C. After 10 ​min incubation at room temperature 150 ​μL of E Lysis buffer was added (60 ​ml potassium acetate, 11.5 ​ml acetic acid and 28.3 ultrapure H2O). Then centrifuged at 14,900 ​g for 5min at 25 ​°C. 700 ​μl of supernatant were added to an equal amount of isopropanol, centrifuged and washed with 70% ethanol, centrifuged at 14,900 ​g for 5min, dried, and suspended in 20 ​μl of ultrapure H2O.

### 2.1 Spectrophotometric detection of DNA concentration

The acquired DNA was diluted (1 ​μl of DNA ​+ ​99 ​μl of 1 ​× ​TE buffer), and the absorbance at the wave lengths of 260 ​nm and 280 ​nm was measured with a spectrophotometer. The buffer in which the DNA was dissolved during the isolation was always used as a blank (i.e. most frequently the TE buffer) which might be replaced with distilled water. DNA purity was determined from the absorbance ratio A260/A280.

For primer and probe design, the National Center for Biotechnology Information website (http://www.ncbi.nlm.nih.gov) was searched using the keywords “zygomycete” and “28S ribosomal RNA sequence” to identify available 28S ribosomal sequences in the Zygomycetes class. Data for real time qPCR probe construction were also supplemented by sequence analysis of PCR products from culture-confirmed Mucorales isolates. The sequences were further examined using OligoPrimer analysis v.6.61 software (Molecular Biology Insights,Inc., Colorado Springs,CO,USA), Primer Express v.3.0 (Applied Biosystems Cheshire,UK), BioEdit sequence alignment editor v. 7.0 software (Isis Pharmaceuticals,Inc.,Carlsbad,CA,USA), and Sequencher v.4.0.5 software (Gene Codes, Inc., Ann Arbor,MI,Italy) to identify suitable regions for primer and hybridization probes based on sequence homologies among five genera (Rhizopus, Lichtheimia, Mucor, Rhizomucor, Cunninghamella). The PCR mixture consisted of 1 ​× ​Master Mix (Platinum qPCR supermix-UDG with ROX, Lifetech,USA), 0.3 ​μM forward and reverse primers, 0.3 ​μM 6-FAM labeled probe, for Rhizopus (RhizoF 5′-TCAGGTTGTTTGGGAATGCA-3’; RhizoR 5′-GGTTTCTCGCCAATATTTAGCTTT-3’; RhizoP 6FAM-CCTAAATTGGGTGGTAAAT-MGB); 0.3 ​μM forward, 0.6 ​μM reverse primers, 0.3 ​μM 6-FAM labeled probe, for Mucor (RhizoF 5′-TCAGGTTGTTTGGGAATGCA-3’; MucorR 5′-GGTCTCTCGCAAATATTTAGCTTT-3’; RhizoP 6FAM-CCTAAATTGGGTGGTAAAT-MGB); 0.3 ​μM forward, 1.35 ​μM reverse primers, 0.4 ​μM NED labeled probe. PC-BioMole-101 positive control were obtained by BioMole, Turin, Italy. Real Time quantitative PCR TaqMan MGB (RT-qPCR TaqMan MGB) amplification and amplicon detection using extracted DNA template was performed using a 7500 Real Time PCR System (Lifetech, Carlsbad,CA,USA). Each reaction mixture contained 18 ​μl of the PCR master mix plus 2 ​μl of the extracted DNA from specimens or control material. The amplifications were run in a 96-well plate at 95 ​°C for 10 ​min, followed by 40 cycles at 95 ​°C for 15 ​s and at 60 ​°C for 1 ​min.

Anova test was used to compare the amount of fungal DNA and genome levels of Mucolares of each extraction methods. Statistical analyses were done using the Prism software (GraphPad Software, La Jolla, CA). In all analyses, p ​< ​0.05 was taken to be statistically significant.

## 3 Results

Five methods for the extraction of fungal DNA from standard and clinical strains were tried out. The main criteria for evaluating the methods used in DNA isolation moulds were the purity and amount of the DNA acquired. The isolation methods should provide a sufficient amount of pure DNA which can be further amplified by real-time PCR. For this purpose, five method of DNA extraction was optimized and compared. The amount of DNA acquired was always related to the original amount of mycelium used for DNA isolation. Each method for DNA isolation was simultaneously used for 6 *Mucorales* clinical strains (*3 Mucor* spp. *and 3 Rhizopus* spp.) for reasons of the varying extractability of their DNA. In our study, five methods were used to evaluate DNA concentration and semi-quantitative determination of the amount of DNA by real-time PCR.

**Fig. 1:**
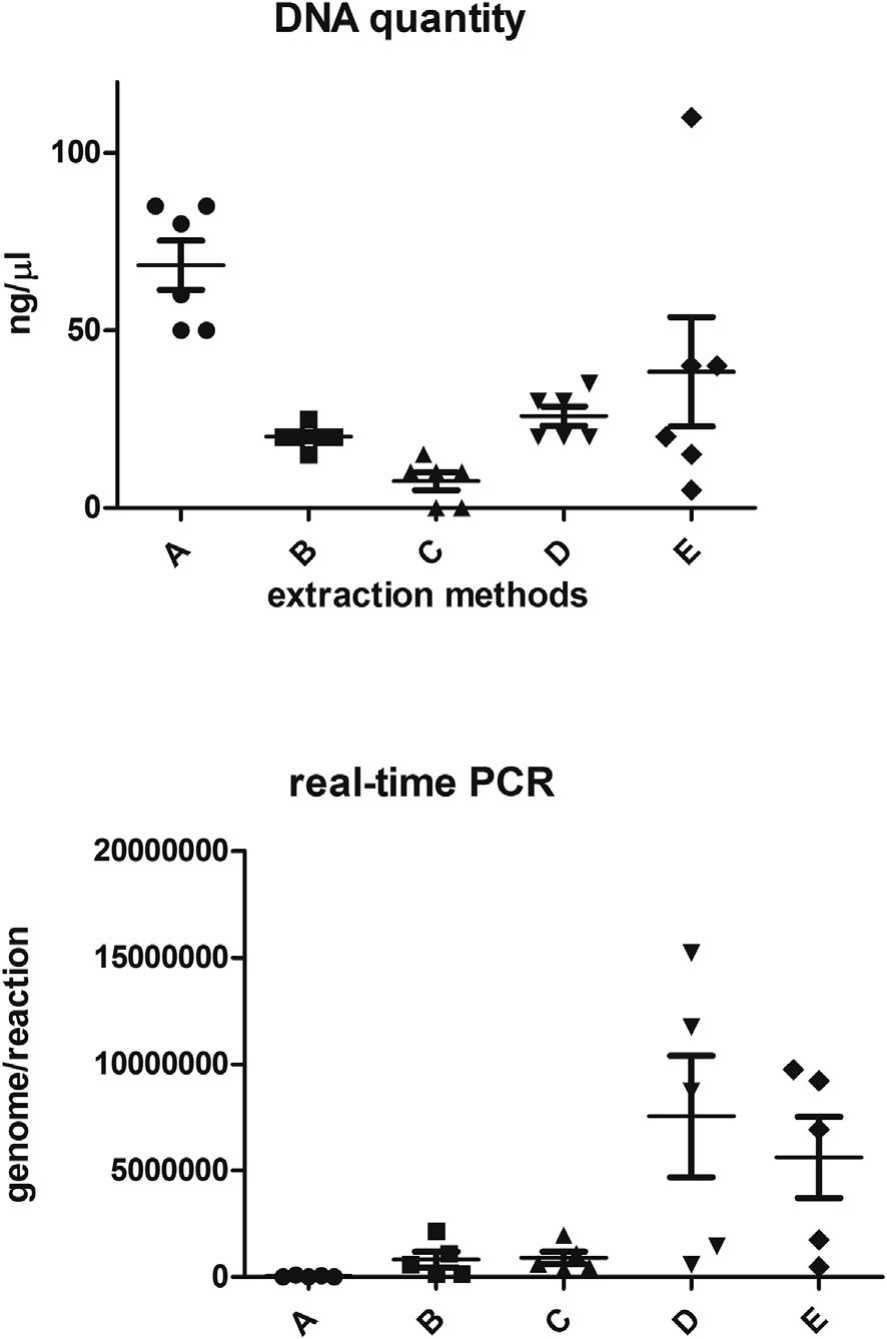
**A)** Anova followed by Kruskall Wallis post-test (ng/μl x extraction methods) revealed a significant difference in term of amount of fungal DNA extracted in five methods studied; **B)** Anova followed by Kruskall Wallis post-test (genome x extraction methods) revealed a significant difference in term of amplificability of fungal DNA extracted in five methods studied.

Level of fungal DNA recovered with five extraction methods are displayed in Table 1. From the viewpoint of concentration and purity, methods A shown abundant amount of fungal DNA whereas methods E report a pure fungal DNA with R260/280 of 1.7 near the optimal 1.8. The DNA quantity reach statistically difference at ANOVA test with p value 0.0005 (Fig. 1a) (see Table 2).

**Table 1:** DNA concentration.

Extraction methods	A		B		C		D		E	
SAMPLES	ng/μl	R 260/280	ng/μl	R 260/280	ng/μl	R 260/280	ng/μl	R 260/280	ng/μl	R 260/280
1	60	1.64	25	2	15	2	35	2.5	40	1.6
2	50	1.67	20	1.3	10	1.3	30	2.5	40	2
3	85	1.4	20	1.3	10	1.3	30	2.5	110	1.5
4	85	1.45	20	2	0	1.5	20	2	20	2
5	50	1.43	20	1.5	0	1.5	20	2	15	1.5
6	80	1.4	15	1.5	10	1.5	20	1.7	5	1.5
**mean**	**68.3**	**1.5**	**20**	**1.6**	**7.5**	**1.5**	**25.8**	**2.2**	**38.3**	**1,7**
**DS**	**17**	**0.12**	**3.16**	**0.32**	**6.1**	**0,25**	**6.64**	**0,31**	**37.7**	**0,24**

**Table 2:** Genome/reaction obtained with *Mucorales* specific real-time PCR.

Extraction methods	1	2	3	4	5	6	mean	ds
**a**	4000	599966	1964192	8724444	6924444	7724884	4553262	3935062
**b**	5539	137952	365968,7	578574,1	475574,1	566574,1	312721,6	237138,8
**c**	95290	2152222	456635,1	11740000	9740000	11747700	4836829	5490225
**d**	64825	1085727	1094172	15211111	9211000	15210000	5333367	6640343
**e**	7223	141451	610931,8	1448469	1748469	1448000	791308,8	777440,8

DNA that could be to amplified was acquired using almost all the methods for isolating DNA. The capacity for DNA amplification was verified by semi-quantitative real-time PCR as described in materials and methods section. Method D shown very high genome number such as E method. The DNA amplificability reach statistically difference at ANOVA test with p value 0.0032 (Fig. 1b).

## 4 Discussion

Commonly, obtaining DNA from fungi is more difficult than from bacteria or from mammalian cells as the fungal cell wall is tough to break using the conventional extraction methods employed for bacteria or viruses. This is attributed to fungi possessing a thick and complex cell wall resulting in poor release of DNA. Poor efficiency of DNA extraction, low numbers of fungal cells in clinical material may be the reasons for obtaining false negative results from clinical specimen [12]. Over the last two decades, many molecular methods have been extensively tested for the detection of fungal DNA in blood and in other clinical samples [13]. Then, in addition to using specific primers and probes, suitable for the detection of low level of fungal DNA, it is particularly important to use the most efficient method of DNA extraction. Additional procedures leading to disruption of the fungal cell wall such as mechanical, enzymatic and/or chemical methods are required [12]. International Society of Human and Animal Mycoses recommending the use of PCR in the diagnosis of mycoses, and agree that extraction procedure is the main factor limiting the efficiency of PCR [14]. Many protocols for the extraction of fungal DNA from clinical specimens employing different commercial kits have been described in the medical literature [12,15]. Griffiths et al. have reported extraction with a Qiagen kit utilizing bead beating technique along with a lysis buffer where they found the yield of DNA better than other methods [12]. Van Burik et al. evaluated the use of glass beads with vortexing for extended periods and found it better for extraction of DNA from filamentous fungi [15]. Currently, the available kits are intended for isolation of human DNA from clinical samples require additional pretreatment for fungal cell disruption.

Here, we have described methods that can be employed in a routine laboratory and that do not involve either freezing using liquid nitrogen or grinding. In our study, the method A based on boiling moulds obtained a greater amount of fungal DNA but failed to extract amplifiable DNA from *Mucorales*. Microwave and freezing, peculiarity of methods B and C respectively, have achieved poor results both in terms of DNA concentration and of amplificability. On the other hand, method E based on addition of a potassium acetate buffer in the lysis step, obtained a decent level of amount of fungal DNA with greater amplifiable DNA from Mucorales. Probably this fact is due to the properties of potassium acetate buffer, able to precipitates sodium dodecyl sulfate (SDS) and SDS-bound proteins to allow their removal from DNA. The acetic acid neutralizes the pH, allowing the DNA strands to renature this allows the DNA not to fragment in the following phases of the process such as vortexing and centrifugation. While the phenol chloroform extraction method is cheaper, it failed to extract amplifiable DNA from some of the filamentous fungi and yeast (Rhizopus spp. and C. neoformans) [16]. Moreover, phenol being a toxic chemical is nowadays avoided in routine practice. The time taken by every kit method was similar to each other. Costing of all the kits were similar. Overall, the E method was the most efficient method in the extraction of DNA from fungal cultures compared to the other methods considering time, cost, technical expertise, and instrumentation. Use of this assay will allow researchers to obtain DNA from fungi quickly for use in molecular assays that previously required specialized instrumentation, was time-consuming or was not conducive to batch processing. The proposed methods are manually protocols but they would be automated using any liquid handler that could be programmed for this. Further evaluation on clinical samples is required in order to check the efficacy of the extraction.

## Funding

The authors would like to express sincere thanks to 10.13039/501100006692University of Turin to fund the Project, BERM_RILO 2018.

## Declaration of competing interest

All authors declare no conflict of interest.
